# The missing focus on women’s health in the First 1,000 days approach to nutrition

**DOI:** 10.1017/S1368980020003894

**Published:** 2021-04

**Authors:** Mai-Lei Woo Kinshella, Sophie E Moore, Rajavel Elango

**Affiliations:** 1 Department of Obstetrics and Gynaecology, BC Children’s and Women’s Hospital and University of British Columbia, Vancouver, Canada; 2 Department of Women & Children’s Health, King’s College London, London, UK; 3 MRC Unit, The Gambia at the London School of Hygiene and Tropical Medicine, Fajara, The Gambia; 4 Department of Pediatrics, BC Children’s and Women’s Hospital and University of British Columbia, Vancouver V6H 3V4, Canada; 5 School of Population and Public Health, University of British Columbia, Vancouver, Canada

**Keywords:** Maternal and child nutrition, First 1,000 Days, Women’s health, Gender inequities

## Abstract

The First 1,000 Days approach highlights the time between conception and a child’s second birthday as a critical period where adequate nutrition is essential for adequate development and growth throughout the child’s life and potentially onto their own offspring. Based on a review of relevant literature, this commentary explores the First 1,000 Days approach with a maternal lens. While the primary objective of the First 1,000 Days approach to nutrition is to reduce child malnutrition rates, particularly chronic undernutrition in the form of stunting, interventions are facilitated through mothers in terms of promoting healthy behaviours such as exclusive breast-feeding and attention to her nutritional status during pregnancy and lactation. Though these interventions were facilitated through women, women’s health indicators are rarely tracked and measured, which we argue represents a missed opportunity to strengthen the evidence base for associations between maternal nutrition and women’s health outcomes. Limited evidence on the effects of dietary interventions with pregnant and lactating mothers on women’s health outcomes hinders advocacy efforts, which then contributes to lower prioritisation and less research.

A growing body of literature points to how sufficient nutrition during the First 1,000 Days, from conception to the child’s second birthday, is essential for proper brain development, healthy growth and immune system that impacts the entirety of the child’s life and beyond^(1–5)^. In 2019, 144 million children under five were stunted, including 29·1 % of children in Africa and 21·8 % of children in Asia, which is indicative of chronic undernutrition within the first 1,000 days of life^(5)^. Stunting is associated with cognitive impairment, reduced potential in school and increased risk of chronic disease later in life^(6–10)^. Additionally, malnutrition is intergenerational, as daughters of malnourished mothers are more likely to have low birth weight (LBW) children^(11,12)^.

The 2008 Maternal and Child Undernutrition Lancet series advanced the idea that childhood stunting was the best measure of malnutrition and its long-term costs to society^(10)^. Coined during a meeting with several development groups hosted by the World Bank in April 2010, the ‘First 1,000 Days’ mobilised the findings of the 2008 Lancet series to build momentum and advocacy in international development and global health^(7)^. The subsequent 2013 Lancet Series on Maternal and Child Nutrition further demonstrated early undernutrition as a serious hidden cause of child mortality^(3)^, increased risk of adult chronic diseases^(13)^ and described building momentum with Scaling Up Nutrition programmes^(14)^. The Scaling Up Nutrition movement represents a commitment by sixty-one low- and middle-income countries and four Indian States (as of 2020) to meet the Sustainable Development Goal Two of eliminating global hunger by 2030 by reducing stunting through focusing on the First 1,000 Days window^(15–17)^.

However, with a focus on child health benefits, maternal nutritional status in terms of underlying malnutrition and women’s health outcomes lack explicit focus, such as maternal mortality, morbidities and well-being indicators. Though First 1,000 Days nutrition-specific and nutrition-sensitive interventions are largely facilitated through pregnant and lactating mothers, women’s health indicators may not be tracked and measured, which we argue represents a missed opportunity for developing interventions that are purposefully designed to improve both maternal and child health.

## Maternal nutritional status and health outcomes

Maternal malnutrition is widely prevalent globally. A systematic review found that average intakes of protein, fat and total energy intakes were lower among pregnant women in low- and middle-income countries than in high-income countries and were frequently insufficient in a number of micronutrients, for example, folate, Fe, Ca and Zn^(18)^. Two linked reviews also revealed consistently suboptimal intakes of folate, Fe, vitamin D, fibre and energy among pregnant mothers in high-income countries^(19,20)^. Maternal undernutrition contributes to adverse effects in both mothers and infants including increased caesarean delivery rates and risk of maternal mortality in mothers and intra-uterine growth restriction in infants, which subsequently increases the risk for birth asphyxia and neonatal infections^(8,21)^. Low maternal BMI and anaemia are also associated with lethargy, reduced physical activity, diminished work capacity and increased risk for maternal mortality^(21)^.

Simultaneously, obesity is also prevalent in low- and middle-income countries and high-income countries, particularly among economically poor populations^(22–25)^. Maternal obesity is associated with serious adverse pregnancy outcomes including early pregnancy loss, preterm birth, stillbirth, gestational diabetes, pre-eclampsia and other hypertensive disorders, higher rates of labour induction and caesarean delivery, as well as associated with childhood obesity^(26–31)^.

## Maternal nutritional interventions

Nutrition-specific interventions and programmes include micronutrient supplementation and fortification, optimal exclusive breast-feeding and complementary feeding practices, and disease prevention and management to directly impact maternal, infant and child health outcomes^(32)^. While the primary objective of the First 1,000 Days approach to nutrition is to reduce rates of stunting among children, mothers play an important role in facilitating interventions through strengthening her nutritional status during pregnancy and lactation. For example, a systematic review of thirty-two observational studies highlighted the potential of preconceptional and first trimester vitamin and mineral supplementation to reduce the risk of neural tube defects, LBW and preterm deliveries^(33)^, while a number of maternal multiple micronutrient (MMN) supplementation trials found significant reductions in LBW^(32,34,35)^.

Nutrition-sensitive interventions also highlight the importance of underlying factors that indirectly impact maternal and child nutrition, such as women’s empowerment^(32,36,37)^. Women’s empowerment, the process of increasing agency and the status of women, influences household access to resources including allocation for child’s health and nutrition^(36)^. Four systematic reviews in the past 4 years evaluated the association between women’s empowerment and child nutrition. They showed promise for improving child health outcomes but described methodological challenges in various definitions of empowerment^(36,38–40)^. While the value of women’s empowerment is acknowledged intrinsically to be beneficial to women and as a means to improve household nutrition, the reviews focused on the latter.

## A gap in measuring women’s health and nutritional status

Though the interventions were facilitated through maternal nutrition, the measurement of women’s health outcomes was overlooked. For example, a large MMN supplementation trial in Tanzania conducted with 8468 HIV-negative pregnant women focused on child health outcomes, with LBW, prematurity and fetal death as the primary outcomes measured^(35)^. The trial, which found that supplementation significantly reduced the risk of LBW potentially due to lower risk of maternal anaemia, also followed a subset of children up to 11–14 years old. Follow-up on women’s health outcomes was not reported, even as potential confounders for child development outcomes^(41)^. More recently, the ‘Women First’ Trial with 7387 women in Guatemala, India, Pakistan and the Democratic Republic of the Congo found that periconceptual maternal MMN supplementation improved fetal growth-related birth outcomes^(42)^. However, a recent Editorial noted the lack of maternal indicators measured in this trial including measures of maternal nutritional status, maternal morbidities, mental health and overall well-being^(43)^. A 2017 Cochrane review of MMN supplementation for women during pregnancy included sixteen trials that reported effects on preterm births and LBW and fifteen trials that reported small-for-gestational age but only five trials reported maternal anaemia, four trials reporting caesarean section rates, three that reported maternal mortality rates and one trial that reported pre-eclampsia^(44)^. Other maternal health outcomes (placental abruption, premature rupture of membranes, maternal well-being or satisfaction) were not reported by any of the trials. This represents a measurement gap in the evidence base particularly in health outcomes for women.

Nutritional interventions focused on the First 1,000 Days have not had the magnitude of effects on reducing childhood stunting as expected. Even with increases in the global rates of exclusive breast-feeding in the first 6 months to 44 % in 2019, rates of stunting remain persistently high at approximately 1 in 5 children (21·3 %)^(5)^. In a specific example from rural Gambia, four decades of intensive health and nutritional interventions helped to halve childhood undernutrition rates but stunting remained persistently high at 30 %^(45)^. Some have suggested that highly publicised maternal and child initiatives, such as ‘safe motherhood’ and ‘child survival’, may not have had the expected impact because too little attention has been given to the nutritional status of women, including mothers^(46)^ and the challenges they face in access to quality nutrition and care throughout their lives^(47)^. Mora and Nestel highlight the need for biologically meaningful indicators to screen undernutrition in women beyond Hb for anaemia, BMI and mid-upper arm circumference, which are inadequate for understanding micronutrient deficiencies^(47)^. In addition, attention to monitoring health outcomes in women is also lacking; for example, the follow-up for the maternal micronutrient study in Tanzania^(41)^ and the Women First trial that overlooked measuring outcomes in women^(42,43)^.

## The vicious cycle of the research-advocacy gap

While the importance of maternal nutrition for the mothers may be implicitly understood in interventions within the First 1,000 Days approach, the missing explicit focus has led to a lack of evidence-based data. Christian and colleagues highlight in their review that the role of poor nutritional status during pregnancy and risk of maternal mortality are ‘unrecognized and underappreciated’ due to lack of funding and sufficient data to explore the association^(32)^. The research-advocacy gap is a vicious cycle where limited knowledge leads to lower prioritisation, which equates to less research on the topic.

Many of these themes are similar to the issues raised by the Safe Motherhood Initiative pioneers in the late 1980s and early 1990s. Launched in 1987, Safe Motherhood Initiative was developed to counter the relative neglect of women’s health within the attention on child survival and the failure to address maternal health as intrinsically valuable, beyond its impact on child health^(48,49)^. Described as a ‘measurement trap’, limited data mutually reinforced low priority of maternal health^(50)^. Quality maternal health data are required to establish levels and trends, to identify determinants of risk and for evaluating the effectiveness of maternal health programming^(50)^. Consequently, a lack of quality maternal health data is both a result and contributor to the neglect of maternal health as a priority topic. Taken in the context of maternal and child nutrition, the relative gap in knowledge on the impact of maternal dietary interventions on the women themselves makes the topic hard to discuss and advocate for, which then contributes to lower prioritisation and less research. To help fill the current research-advocacy gap, there is a need for research on the impact of maternal nutrition on women’s health and understanding the gendered contexts of food and nutrition security. It is a missed opportunity that maternal mortality and morbidity outcomes, as well as maternal satisfaction, are not routinely measured in nutritional studies within the First 1,000 Days, such as in maternal MMN supplementation trials.

## Conclusion

While the First 1,000 Days approach explores an intergenerational cycle of poverty and malnutrition through impacts on infants, there is also a need to understand the processes of entrenching poverty and malnutrition between inadequacies in maternal diet, adverse health outcomes for women and contextual factors, with the mothers at the centre. While mothers are targeted to facilitate nutritional interventions to improve infant health outcomes, the measurement of health outcomes in women is lacking. There is a great hope and potential for nutrition to strengthen infant growth and the society they will live in, but our vast dreams for the future cannot neglect the potential for benefits for women and her own life in the here and now.
